# Paying Attention – and Respect – to the Agency of Conflict-Affected Health Workers: Comment on "Human Resources for Health in Conflict Affected Settings: A Scoping Review of Primary Peer Reviewed Publications 2016–2022"

**DOI:** 10.34172/ijhpm.9580

**Published:** 2026-02-15

**Authors:** Enrico Pavignani

**Affiliations:** Institute for Law, Politics and Development (DIRPOLIS), Sant’Anna School of Advanced Studies, Pisa, Italy.

**Keywords:** Adaptation, Agency, Crisis, Informality, Politics, Survival

## Abstract

The review stands out for its methodological rigour, clear results, and frank recognition of its limitations. However, the picture proposed by it is incomplete. Two aspects of great consequence are discussed in this commentary as a complement to the review. First, the political agency of human resources for health (HRHs) must always be considered. Among them, many take sides in a variety of roles, overt or not, as militants, activists, supporters, and researchers. Second, without including the informal practices adopted by HRH to survive and deliver in hostile environments, the health labour market cannot be understood. Arguably, these two key dimensions were not prominent in the review because the HRH literature prefers to focus on formal technical aspects easier to study and more likely to be published. Some of the reasons behind their neglect are suggested by this commentary, which concludes with a few remarks about how this drawback might be corrected.

## Introduction

 The review by Onvlee et al^1^ has much to be commended: it presents a broad range of findings, competently extracted from the published literature. It summarises complex issues in a clear way, adding nuances when necessary. It frankly acknowledges the limitations of such an exercise. However, two key aspects are not discussed as they deserve: the political engagement of health workers and their reliance on informal practices, both manifestations of their agency. In fact, health workers are constituents of societies in turmoil, and active participants in adaptive processes.

 In conflict-affected settings (CAS), *laissez-faire* prevails. The boundaries between public and private providers are blurred; skilled and unskilled health workers operate together; training policies are misconceived or unenforced; protracted turmoil affects every aspect of life; and poor regulation cannot address inefficiencies, maldistribution and unacceptable quality of care. Distressed as well as mistrusted, health workers react in a variety of ways, with mixed effects on healthcare provision. A tainted relationship between providers and users is the price to pay for the precarious survival of under-resourced services, operated by health workers as they see fit. In many settings, the privatisation from within has advanced to such an extent that health workers have become the real owners of the services.^2^

 In these contexts, health workers operate across countless uncertainties. They rely on entrenched informal practices that determine the governance of health services and their interactions with formal policies. Such features are not hidden, but well known by stakeholders, who often prefer to consider them as transitory. Official documents and much research ignore “*the real rules of the game.”*^3^ Because of their importance, they should take centre stage, instead of being regarded as fringe concerns studied mainly by social scientists. Recognising the gap between official provisions and actual events is the precondition to conceive sound measures, and to adjust them as their effects – expected as well as unexpected – become apparent.

## Two Aspects Frequently Ignored – or Under-Explored – in the Human Resources for Health Literature

###  a. Politics Shapes Everything, Including Events in the Human Resources for Health Field

 In violent, high-risk and low-trust environments, politics colours every social aspect, including healthcare provision. But much published literature focusses on technical aspects, neglecting the political participation of health workers in the social processes shaking divided societies. During conflict and after it, such a disregard is totally unjustified, pretences of neutrality notwithstanding.^4^

 Howe and Stites^5^ have described in vivid terms the existential engagement of field workers: *“For local actors and organisations operating in Syria or other insecure contexts, it is personal—it is about their country, their livelihoods, their families, and their future.” “There is nothing ‘remote’ about events in northern Syria for the local organisations included in this study. Their offices are being bombed, their employees attacked, their movements monitored, and their lives are in constant danger. Many are not even receiving salaries.”* Such a description, referred to local humanitarians, is certainly valid for health workers.

 In many post-colonial states, healthcare has been a pillar of the social contract. Challenging authoritarian regimes implies the provision of alternative services, including health-related ones. Therefore, many health workers make clear that they conceive their work as a political act against their foes, who in turn retaliate. Their political affiliation conditions their power, job location, career evolution, and post-conflict prospects. The abundant literature on healthcare provision in opposition-held Syria is informed by this political stance. Such wealth of information contrasts with the dearth of data published on healthcare in regime-controlled areas, which speaks volumes about the ways the respective rumps of the healthcare arena were ruled.

 The identification of healthcare provision and political militancy has a long history in Palestine, where political organisations were banned by the occupiers, whereas health-related ones were partially tolerated.^6^ Such an identification has resisted decades of oppression. In Gaza, many health professionals persist practicing in unbearable circumstances, an astonishing act of survival and defiance.

 Political frontlines condition researchers who cannot investigate healthcare provision in areas controlled by repressive power holders. This hurdle was patent in Myanmar, with much of the published literature focussing on opposition-controlled areas. Moreover, cultural fault lines impede trustful relationships with other professionals and users. See—as an example among many—the fraught dynamics undermining healthcare provision in Kosovo.^7^

 Most political manifestations occur in informal spaces created or expanded by the crisis.^8^

###  b. Informal Practices Are Recognisable in Every Healthcare Arena

 In CAS, they are exposed to a greater extent. To name some of the aspects to be looked at: the transactions between providers and users, the posting of health workers, the assignment of tasks in the workplace, the complex remuneration of health workers and pharmaceutical supplies. Rather than constituting parallel systems, informal practices are an integral component of healthcare provision. In their absence, services would stall. Whereas the coping strategies adopted by human resources for health (HRHs) have been studied (mostly under the heading of corruption), the informal practices aimed at delivering health services in forbidding conditions have been poorly documented.

 The multiple informal measures adopted by health services to recruit unemployed staff, and in turn the ways these workers complement their low wages, are a case in point. *Under-/unemployment* is induced by over-production against limited absorption, and/or unwillingness of professionals to fill existing vacancies. Additionally, poor-quality training reduces the job-finding prospects of new graduates. The shrinking of public revenues induced by conflict depresses employment, be it through frozen recruitment, real-term contraction of wages, or their delayed payment.

 The recurrent complaint about staff shortages is frequently based on counts of public employees, gauged against notional international benchmarks. In this way, unemployed or informally employed health professionals are *“understudied and underacknowledged.”*^9^ In Sudan, unemployment ranked among the main causes of outmigration,^10^ as it is the case in Yemen. Unemployment was a vast and growing phenomenon in Palestine.^11^ In Sierra Leone, half the health workers active at PHC level were not included in the payroll.^12^

 The harm caused by informal practices is usually emphasised to justify measures intended to suppress them: commoditisation of healthcare weakening its quality and causing iatrogenic effects, cost inflation reducing access for the poor and service fragmentation hindering referrals, to name just a few manifestations. However, the benefits of informality in distressed healthcare contexts should also be acknowledged. Some of such benefits allow health services to be delivered amidst crushing constraints, such as:

- *Keeping health staff in their workplace*, sometimes in jobs discordant with their qualifications.^13^
- *Providing services or negotiating arrangements discreetly*, where openness would be dangerous. In the memorable expression of an informant on northwest Syria: *“‘frontline health workers’ become ‘undercover relief workers.’”*^14^
- *Offering services otherwise unavailable*, such as home care (a preferred delivery modality where health facilities are targeted by bombers) or outside opening hours.
- *Circumventing unfair or inapplicable norms* or evadingunrealistic donor demands. See Honein-AbouHaidar et al^15^ for the instructive example of Syrian health professionals in Lebanon.
- *Delivering services in the absence of formal interventions*. The grassroots response to the 2023 earthquake – enabled by the experience gained by stakeholders over many years of crisis management – exemplifies the power of trust-based informal networks in Northwest Syria.^16^

Other benefits are reaped by users, who may adopt informal practices to:

- *Escape the sight of abusive or violent state agents* and the toll extracted by them.
- *Shorten or bypass cumbersome procedures*, when a delay might be costly or harmful.
- *Obtain information about options and costs*, which influences health-seeking behaviour.

## Why Are Such Central Aspects Regularly Ignored by Conventional Studies?

 Politics is left aside as contentious, to pre-empt the reactions of warring parties seeing healthcare through a security lens, or of authoritarian governments eager to parade their successes. In their review of research on a highly praised public health programme in Ethiopia, Østebø et al^17^ found that the political context was marginalized. A host of factors, both global and specific to the country, contributed to this research gap. These authors concluded that the silence about political issues is proof of the importance attached to public-health interventions by repressive authorities.

 Data undisclosed by health authorities rarely reach researchers, who prefer to investigate more conducive settings. Perversely, studies recognising the lack of solid data on important issues are rarely written and published. Because omissions are information, sometimes more valuable than disclosed data, focussing on them may provide precious insights. On the same vein, studies challenging fishy data released by official health authorities are rare. They should be encouraged by both funding bodies and academic journals.

 A major knowledge gap in the published literature relates to the ways found by frontline workers to manage risks, confront unforeseen events and act outside their official duties, in order to serve needy populations.^14^ Wary of incurring in donor penalties for evading their contractual obligations, implementers avoid reporting about such practices. Instead of providing insight or inspiration, such under-the radar solutions or workarounds may therefore be overlooked. But without mutual trust, a scarce asset rarely won over by outsiders, communication is hindered.

 The conviction that evidence is mostly generated through controlled experiments, which are unfeasible or unreliable in messy, fluid contexts, leads to downplaying the insights provided by other sources of knowledge. Thus, quantitative data, much easier to obtain in relation to formal employees, are preferred to qualitative ones. The preference of many reviewers for papers following such orthodoxy discourages authors to adopt heterodox perspectives.^18^ In light of these considerations, the peer-reviewed literature tends to represent the orthodoxy, not necessarily the strongest evidence, or the most useful one.

## Studying Human Resources for Health as They Behave, Rather Than as They Should Do

 By sticking to official data, many analyses of conflict and post-conflict settings ignore the bulk of the workforce. This is constituted by informal providers lacking official qualifications, unsalaried health professionals working in public facilities and qualified health workers preferring to work at the margins of regulations. Methods developed to study the health labour market in stable, controlled settings tend to miss these categories of workers, or to misrepresent them.

 Alternative ways, such as realist^19^ and interpretive^20^ reviews, may be better suited to explore issues such as informality or hidden systems, which are may only be hinted at, or mentioned as an aside. By assembling the insights offered by several studies, chosen for their relevance rather than their methodological rigour, complex issues can be illuminated. In their diversity, findings may converge towards compelling interpretations. Particular weight should be given to any finding dissenting with dominant narratives, which “…*should be valued as an opportunity rather than dismissed as an anomaly.*”^21^

 If the review is silent about the two dimensions discussed above is not for lack of effort by the authors. It seems that the strict inclusion criteria adopted by the review undermined its breadth and depth. Including grey documents would improve any review, if the strengths and weaknesses of these materials are taken into accounts. Their contents should be interpreted in appropriate ways. Grey documents offer some advantages over peer-reviewed publications. They are often written by practitioners concerned with actions on the ground and related to recent events. They may be franker about the dilemmas faced in the field. Importantly, they may describe negative outcomes, which are usually ignored by the published literature. On the negative side, grey documents may be methodologically weak and often sanitise facts in order to satisfy donors. Ideally, an interaction between scholars and implementers might enhance the interpretation of reality.^22^ Checking the quality of grey documents entails further work by researchers, but such an effort may provide rewards.

 Concluding, carrying out a study of HRH in CAS with broader inclusion criteria might constitute a stimulating experiment: what additional insights might be gained from including (*a*) grey literature, (*b*) documents in French, Russian and Arabic, (*c*) studies on crisis settings (not just conflict-affected ones), (*d*) papers covering a broader time span, and (*e*) analyses of whole health systems providing important clues about health workers? And which insights obtained from an orthodox selection might be challenged by an expanded one?

## Disclosure of artificial intelligence (AI) use

 Not applicable.

## Ethical issues

 Not applicable.

## Conflicts of interest

 Author declares that he has no conflicts of interest.
